# Small molecules that enhance mitophagy to delay aging and neurodegeneration

**DOI:** 10.20517/jca.2022.36

**Published:** 2022-09-13

**Authors:** Gerald W. Dorn

**Affiliations:** Center for Pharmacogenomics, Department of Internal Medicine, Washington University School of Medicine, St. Louis, MO 63110, USA.

Mitochondria are central arbiters of cell fate. Mitochondrial respiration produces ATP, the chemical fuel for most biological processes in multicellular organisms. Conversely, mitochondrial respiration that is not properly coupled to ATP synthesis produces mito- and cytotoxic reactive oxygen species (ROS) that can damage cell and organelle DNA, protein and lipid, thereby evoking premature cell senescence or programmed death. The dual role of mitochondria as sustainers of cell life vs engines of cell death requires cells to deploy surveillance and removal systems that identify, isolate and selectively destroy dysfunctional mitochondria that, if retained, would pose a threat to cell wellbeing. The principal process by which overall mitochondrial quality is maintained is through selective culling of dysfunctional and damaged organelles by mitochondrial autophagy, or mitophagy. It is posited that age-related deterioration in mitophagy, and the consequent interruption of mitochondrial quality control, can contribute to adverse aging phenotypes partly because of increased elaboration of mitochondria-derived ROS from improperly retained damaged organelles^[1]^. A corollary to this hypothesis is that improving the overall fitness of the cellular mitochondrial collective by forced mitophagy activation might delay age-related cell degeneration and ameliorate age-associated diseases. Experimental systems using overexpression of mitophagy and related factors support this proposition^[2]^. However, “gene therapy” is not readily translatable to the clinic in this context. Thus, a clinically applicable therapeutic approach to enhance mitophagy is much needed. Recently, Einav Gross and colleagues took a significant step toward achieving this goal by developing small molecule derivatives of spermidine that exhibit mitophagy-enhancing activity in *C. elegans* and cultured mammalian cells^[3]^.

In their manuscript, “Distinct designer diamines promote mitophagy, and thereby enhance healthspan in *C. elegans* and protect human cells against oxidative damage”^[3]^, senior author Gross, first author Srivastava, and co-authors build upon previous observations that the aliphatic polyamine spermidine, commonly used in molecular biology laboratories as a DNA binding agent, can delay death by enhancing autophagy^[4]^. The chemical structure of spermidine is straightforward, consisting of an 8-member aliphatic chain linking amines at each end, with a third amine offset in the middle [Figure 1]. Srivastava and co-workers aimed to develop chemical analogs with enhanced potency or other pharmaceutical advantages and use them in the laboratory to better understand how aliphatic polyamines increase autophagy/mitophagy. Toward this end, chemical variants of spermidine were synthesized and screened for enhanced mitophagy activity. The lead compound, VL-004, differs from spermidine only by the absence of the middle linker amine [Figure 1], but it has improved mitophagy-enhancing activity and conferred superior protection against ROS. Detailed studies comparing VL-004 to spermidine and its other derivatives revealed the following major findings:

1. VL-004 increases mitophagy, measured as mitochondrial delivery to lysosomes and mitochondrial DNA content, in *C. elegans* skeletal muscle and neurons.
2. Mitophagy enhancement by the polyamines requires the worm analogs of mammalian Nix/Bnip3 and PINK1 kinase, but not the canonical mitophagy effector, Parkin.
3. Mitophagy activation by VL-004 requires, induces, or is otherwise impacted by altered expression of aging and autophagy genes.
4. VL-004 increases longevity (survival benefit) and improves physical activity (quality of life benefit) in older worms.
5. Too much mitophagy has adverse consequences, defining a therapeutic window for mitophagy activation.

These five major findings, and many others in this highly detailed report, provide fodder for a fresh examination of the relationships between mitochondria and normal senescence or age-related disease. The discussion below uses the mammalian designations for the various factors that Srivastava *et al.* studied primarily in worms^[3]^.

Mitophagy is an example of biological multi-tasking, deploying components of the cell starvation autophagy stress-response for the largely homeostatic process of surveilling and removing “mitochondrial trash” (i.e., senescent, damaged or degenerated mitochondria) before it interferes with normal cell functioning^[5]^. The most familiar mitophagy activation pathway is mediated by two juvenile Parkinson’s disease gene products, PINK1 kinase that serves as a trigger for mitophagy in individual dysfunctional mitochondria, and the Parkin ubiquitin ligase that amplifies the mitophagy response at targeted mitochondria. In this selective mitochondrial culling process, PINK1 kinase is protected from proteolytic degradation, enabling its phosphorylation of mitofusin (MFN) 2 and other mitochondrial Parkin receptor proteins that recruit Parkin from its cytosolic storage site to the damaged organelle^[6]^. Parkin poly-ubiquitination of multiple mitochondrial outer membrane proteins, using PINK1-phosphorylated ubiquitin as the preferred substrate, attracts cellular autophagosomes that engulf the degenerating mitochondrion and transfer it to lysosomes for destruction^[7]^. This terminal event of mitochondrial transference to lysosomes is the *sine qua non* of mitophagy, and was the primary metric of mitophagy that Srivastava *et al.* measured in transgenic worms expressing a mitochondrial-targeted green/red fluorescent protein chimera that changes fluorescence characteristics when mitochondrial proteins are introduced into the acidic environment of the lysosome^[3]^.

Mitochondrial quality control is the canonical role for mitophagy, and likely the mechanism by which its enhancement prevents premature senescence. However, culling defective mitochondria is not the sole purpose of mitophagy, nor is the PINK-Parkin pathway the only pathway to mitophagy. Accumulating evidence in mammalian systems suggests that Parkin-mediated mitophagy may be more important as a stress-induced or developmentally regulated mechanism, and that other paths comprise the major mechanism for homeostatic quality control through “maintenance mitophagy”. Moreover, Parkin-mediated mitophagy can play an essential role during cell-wide mitochondrial replacement, generally accelerating mitochondrial turnover for either quantity control (i.e., removing excess mitochondria) or provoking a metabolic adaptation in response to an altered environmental context (as in converting the mitochondrial collective from lipid- to carbohydrate-based metabolism and vice versa)^[8]^. Indeed, while Srivastava *et al.* report that PINK1 kinase was critical for mitophagy enhancement by their novel small mitofusin activating molecules^[3]^, Parkin was dispensable. Rather, the worm analog of Nix (Bnip3L)/Nip (Bnip3) mitophagy adaptor proteins was required, pointing to mitofusin activation by one of the Parkin-independent pathways. This is consistent with spermidine-derived small molecule mitophagy activating compounds augmenting, in large part, constitutive/homeostatic mitochondrial quality control.

Mitochondrial abnormalities such as fragmentation, loss of inner membrane polarization and increased ROS production have been widely reported in both chronically progressive neurodegenerative diseases and genetic neurological syndromes manifesting an age-dependent phenotype^[9]^. In most instances, such as Alzheimer’s disease, Huntington’s disease and genetic forms of amyotrophic lateral sclerosis, the underlying causal genetic mutation is neither encoded by the mitochondrial genome nor affects one of the ~1000 nuclear-encoded mitochondrial proteins. (A notable exception is the progressive peripheral neuropathy, Charcot-Marie-Tooth disease type 2A, which is caused by mutations of the mitochondrial fusion protein MFN2^[10]^). Nevertheless, there is growing interest in developing approaches to correct secondary mitochondrial abnormalities as one component of an ensemble therapy approach to these incurable and largely untreatable diseases. One promising approach attacks the problem of mitochondrial fragmentation, either by inhibiting mitochondrial fission or stimulating mitochondrial fusion. It is intriguing to speculate that these tactics might act synergistically with pharmaceutical activation of mitophagy to correct mitochondrial abnormalities in neurodegenerative diseases.

One unanswered question about VL-004 and related small molecule mitophagy activators is “what is the drug target”? Srivastava *et al.* have taken full advantage of genetic systems to define transcriptional pathways and mitophagy co-factors that mediate enhanced mitophagy, but the actual drug target protein is not known. Information on the VL-004 target and binding characteristics could help direct further chemical modifications to improve potency to the nM range of most orally active pharmaceutical agents.

## Figures and Tables

**Figure 1.**
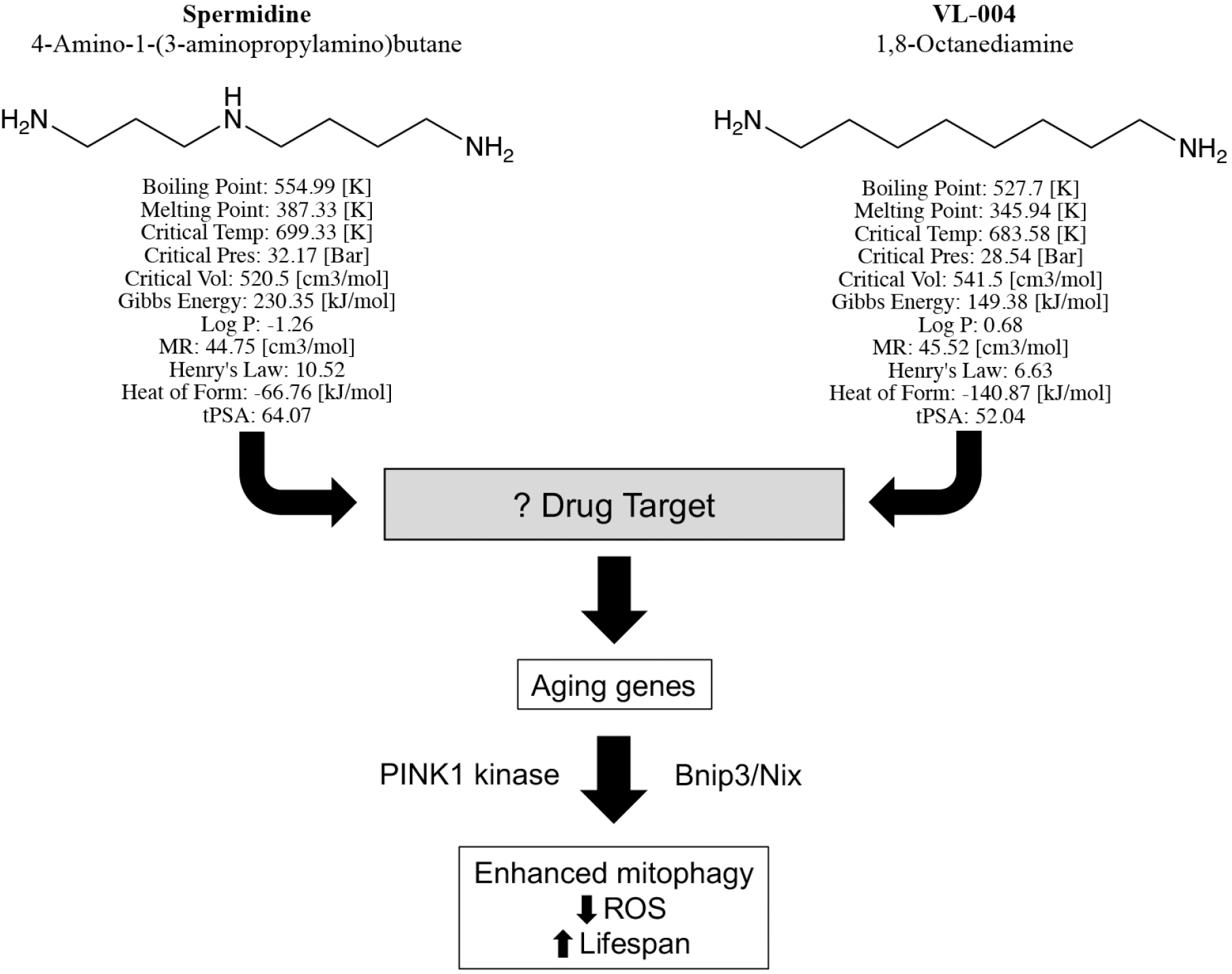
Structures, predicted chemical properties, and mitophagy activating pathway of Spermidine and VL-004, described by Srivastava *et al*. “Log P” is the water octanol partitioning coefficient^[3]^; “MR” is molar refractivity; “Henry’s law” is solubility constant; “Heat of form” (formation) is enthalpy; “tPSA” is topological polar surface area. Neither compound has any exception to Lipinski’s rule, suggesting that they both have pharmaceutical properties of orally active drugs.

## Data Availability

Not applicable.
